# Schizo - obsessive disorder - separate clinical entity or elusive comorbidity? - a systematic review

**DOI:** 10.1192/j.eurpsy.2024.602

**Published:** 2024-08-27

**Authors:** R. Niewiadomski, J. Maciejczyk-Kawecka, M. A. Ciołek, K. Wilczyński, M. Janas-Kozik

**Affiliations:** ^1^Pediatric Centre of John Paul II, Sosnowiec; ^2^Students’ scientific association at the Department of Psychiatry and Psychotherapy of Developmental Age; ^3^Department of Psychiatry and Psychotherapy of Developmental Age, Medical University of Silesia, Katowice, Poland

## Abstract

**Introduction:**

In some clinical scenarios obsessive and delusive symptoms exhibit several similarities, making it challenging to differentiate between schizophrenia spectrum disorder (SSD) and obsessive-compulsive disorder (OCD). There are numerous reports of patients suffering from those disorders and manifesting both psychotic and obsession-like features, which makes accurate distinction even more complicated. We found several conflicting theories attempting to elucidate this overlap, one being the existence of the separate clinical entity - schizo-obsessive disorder.

**Objectives:**

The aim of this study is to consolidate current knowledge, synthesize existing theories and explore diagnostic implications.

**Methods:**

We conducted a systematic literature review following the PRISMA protocol, we scrutinized studies addressing obsession-like symptoms in SSD, psychotic symptoms in OCD, and comorbidity of those disorders. We included peer-reviewed non-interventional studies published in English and Polish from 2013 onwards. The search was performed in the following medical databases: PubMed, Science Direct, Scopus, and Web of Science. Synthesis utilized a narrative approach due to diverse study designs, outcomes and observational nature of the collected data.

**Results:**

We identified several dozen articles, which revealed a range of diverse findings, often inconclusive, and occasionally conflicting, Although, the collected data indicate the schizo-obsessive spectrum exhibits clinical relevance.

**Conclusions:**

The ambiguity in results emphasizes the necessity for further investigations into pathomechanism of schizophrenia and OCD. Future research, particularly involving children and adolescents, should strive for a comprehensive understanding of the nuanced manifestations of obsessive-like and psychotic symptoms in both disorders, aiding in refining diagnostic criteria and developing effective intervention strategies.

**Disclosure of Interest:**

None Declared

